# A new species of *Lipogramma* from deep reefs of Roatan, Honduras (Teleostei, Grammatidae)

**DOI:** 10.3897/zookeys.809.29280

**Published:** 2018-12-19

**Authors:** Luke Tornabene, D. Ross Robertson, Carole C. Baldwin

**Affiliations:** 1 School of Aquatic and Fishery Sciences, Burke Museum of Natural History and Culture, University of Washington, Seattle, WA, USA University of Washington Seattle United States of America; 2 Smithsonian Tropical Research Institute, Balboa, Republic of Panama Smithsonian Tropical Research Institute Balboa Panama; 3 Department of Vertebrate Zoology, National Museum of Natural History, Smithsonian Institution, Washington, DC, 20560, USA National Museum of Natural History, Smithsonian Institution Washington United States of America

**Keywords:** systematics, phylogeny, Caribbean, basslet, species delimitation, submersible

## Abstract

A new species of *Lipogramma* is described from submersible collections at 122–165 m depth off the coast of Roatan, Honduras, in the western Caribbean. The new species is distinguished from all other species in the genus by its bright blue coloration on the head, nape, and dorsal portion of the trunk beneath the spinous dorsal fin, a prominent round black blotch below the origin of the spinous dorsal fin, and a high number of gill rakers. A molecular phylogeny based on mitochondrial and nuclear genes shows that the new species belongs to a clade containing *L.levinsoni*, *L.regia*, and *L.anabantoides*. At Roatan, submersible observations of this and other *Lipogramma* species indicate clear, interspecific habitat partitioning by depth and substrate.

## Introduction

Manned submersibles have proven to be highly effective for collecting fishes from deep-reef habitats (Gilmore 2016), particularly in the rariphotic zone (below ~130 m; Baldwin et al. 2018), where divers using closed-circuit rebreathers, which are limited to depths less than ~ 150 m, are incapable of sampling for extended periods of time. This is especially true for cryptobenthic fishes such as grammatids and gobiids, many of which are associated with structurally complex reef and rocky habitats and are unlikely to be sampled using trawls or dredges. Partially because they are difficult to sample, cryptobenthic reef fishes as a whole are an understudied group, and recent studies suggest that they comprise nearly half of all fish diversity on coral reefs and possess a large number of undescribed species (Brandl et al. 2018). In recent years, researchers from the Smithsonian Deep Reef Observation Project (DROP) have used the manned submersible *Curasub*, located on the island of Curaçao in the southern Caribbean and capable of descending to 300 m, to collect and describe a cache of new species of reef fishes, including many species of cryptobenthic fishes (e.g. gobiids, Baldwin and Robertson 2015, Tornabene et al. 2016a, 2016b, Tornabene and Baldwin 2017; labrisomids, Baldwin and Robertson 2013; and grammatids, Baldwin et al. 2016, 2018).

In 2017, DROP operations expanded to Roatan, Honduras, where the *Idabel* manned submersible is located. On the first dive capturing specimens with *Idabel* off Halfmoon Bay, West End, Roatan, the authors collected a specimen of an undescribed species of *Lipogramma* (Grammatidae) at 165 m depth. Subsequent dives around this depth revealed that the species is relatively common, despite never having been collected before nor observed in more than 150 submersible dives throughout the Caribbean by us or others using *Curasub*, *Idabel*, or the *Johnson Sea-Link* subs. Four additional specimens, including one juvenile, were subsequently collected on later dives off Roatan.

The description of the new species from Roatan brings the total number of species in *Lipogramma* to 13, all of which occur in the tropical western Atlantic Ocean. *Lipogramma* and *Gramma* are currently classified in the family Grammatidae based on a single synapomorphy in the arrangement of cheek musculature (Gill and Mooi 1993). This relationship is not supported by molecular data, although the relationships between genera in the diverse Ovalentaria have proven to be difficult to resolve with traditional molecular markers, a combination of molecular markers and morphological characters, and phylogenomic data (Betancur-R et al. 2013; Mirande 2016; Eytan et al. 2015). Nearly all species of *Lipogramma* occur on deep reefs. *Lipogrammatrilineata* and *L.anabantoides* are the only two species known to routinely occur above 50 m, which is approximately the limit of recreational scuba diving. Including the new species described here, five species of *Lipogramma* have been described from specimens collected using *Curasub* and *Idabel*. Several species are rare in collections, owing to the difficulties of collecting fishes from structurally complex deep-reef habitats. Currently *L.haberorum*, *L.barrettorum, L.schrieri*, *L.robinsi*, *L.flavescens*, *L.regia*, *L.rosea*, and the new species described here are each known from fewer than 10 specimens, although this may not accurately reflect an actual rarity in the wild. Increased sampling from Roatan and other localities across the Caribbean are certain to uncover additional undescribed species of *Lipogramma* and other cryptobenthic fishes.

## Materials and methods

**Figure 1.**
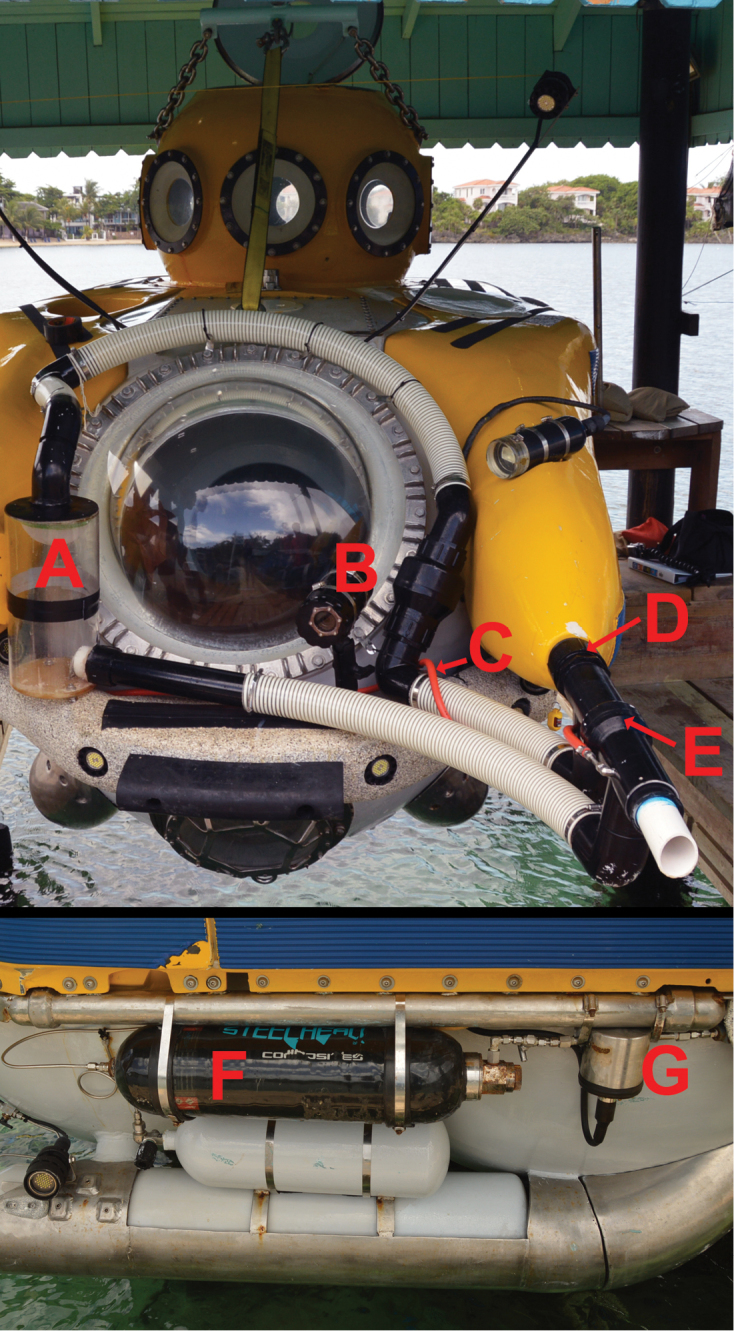
*Idabel* submersible outfitted with fish-catching system. **A** acrylic holding tank **B** housing for HD video camera **C** quinaldine sulphate delivery hose **D** suction for the system is powered by a PVC hose connecting to one of the submersible’s vertical thrusters **E** two-way valve to allow for differential suction/blowing of water and or anesthetic **F** carbon-fiber compensator holding up to 2.5 gallons of anesthetic solution, powered by pressurized air from a SCUBA cylinder (not shown, lower tank in image is oxygen for life support systems) **G** housing for solenoid valve, enabling scientists to control the flow of anesthetic from a switch inside the submersible.

Specimens were collected using the *Idabel* submersible. The *Idabel* can accommodate a pilot and two scientists and is capable of diving to ~700 m. This sub was recently outfitted with a fish-catching system capable of delivering an anesthetic solution (5% quinaldine sulphate in seawater) and capturing specimens with a suction system powered by one of the submersible’s vertical thrusters (Fig. 1). Four of the five type specimens were brought to the surface alive where they were photographed prior to euthanasia in MS222 and preservation. Tissue samples were taken and stored in 95% ethanol, and voucher specimens were fixed in 10% formalin and later transferred to 70–75% ethanol. Measurements were made weeks to months after preservation, and were taken to the nearest 0.1 mm with digital calipers. Counts and measurements follow Hubbs and Lagler (1947). Specimens were x-rayed with a digital radiography system. Type specimens were deposited at the University of Washington (**UW**), the National Museum of Natural History, Smithsonian Institution (**USNM**), the Florida Natural History Museum (**UF**) and the National History Museum of the National Autonomous University of Honduras in the Sula Valley (**MUVS-V**). Cephalic pores were viewed and photographed using a Zeiss Discovery V20 SteREO microscope with an attached Axiocam 503 digital camera. In addition to comparing our morphological data to those from original species descriptions, our comparative material examined here included several specimens (including types) of *L.evides*, *L.levinsoni*, *L.barrettorum*, *L.schrieri*, *L.haberorum*, as well as the voucher specimens from our phylogenetic analysis. Catalog numbers of these specimens are listed in the appendices of Baldwin et al. (2016) and Baldwin et al. (2018).

DNA was extracted from tissue samples using a Qiagen DNeasy Blood and Tissue kit. Four loci were sequenced for three specimens of the new species for phylogenetic analysis. A partial segment of the mitochondrial gene cytochrome c oxidase subunit I (COI), and three nuclear genes (TMO-4C4, Rag1, Rhodopsin) were amplified via PCR and sequenced using primers and PCR conditions from Weigt et al. (2012) and Lin and Hastings (2011, 2013). GenSeq nomenclature (Chakrabarty et al. 2013) and GenBank accession numbers are listed in Appendix 1: Table A1. The sequence data was aligned from the Roatan samples with data from our past studies (Baldwin et al. 2016, 2018). The alignments were concatenated and phylogeny was inferred using Bayesian Inference (BI), partitioning by gene. For the BI analysis, the substitution models and the partitioning scheme were chosen using PartitionFinder (Lanfear et al. 2012) according to Bayesian Information Criterion scores. The BI phylogeny was inferred in the program MrBayes v. 3.2 (Ronquist et al. 2012) using two Metropolis-coupled Markov Chain Monte Carlo (MCMC) runs, each with four chains. The analyses ran for 10 million generations sampling trees and parameters every 1000 generations. Burn-in, convergence and mixing were assessed using Tracer (Rambaut and Drummond 2007) and by visually inspecting consensus trees from both runs. The ML analysis was done in the program RAxML v.8.2.9 (Stamatakis 2014), using 20 initial random searches, and topological support was assessed using 1000 bootstrap replicates. Outgroups for the phylogenetic analysis included two species of *Gramma* and several other genera from the Ovalentaria *sensu*Wainwright et al. (2012), i.e. *Acanthemblemaria* (Chaenopsidae), *Blenniella* (Blenniidae), and *Tomicodon* (Gobiesocidae).

A coalescent-based Bayesian species-delimitation analysis was also conducted (Yang and Rannala 2010, 2014) using the program BP&P ver 3.2 (Yang and Rannala 2010; Yang 2015). This program analyzes multi-locus sequence alignments under the multispecies coalescent model (Rannala and Yang 2003). Each individual was assigned to one of ten groups (nominal species) a priori, based on the potentially diagnostic morphological and pigmentation characters. BP&P was then used to infer a species tree and calculate and compare the posterior probabilities of different species-delimitation models that comprised ten species versus alternative models with fewer than ten (lumping “morpho-species”) or more than ten (splitting “morpho-species”).

## Taxonomy

### Lipogramma idabeli sp. n.

Taxon classificationAnimaliaPerciformesGrammatidae

http://zoobank.org/FC54BAB6-F303-48EC-8F03-E13BAE5534FA

**Figure 2.**
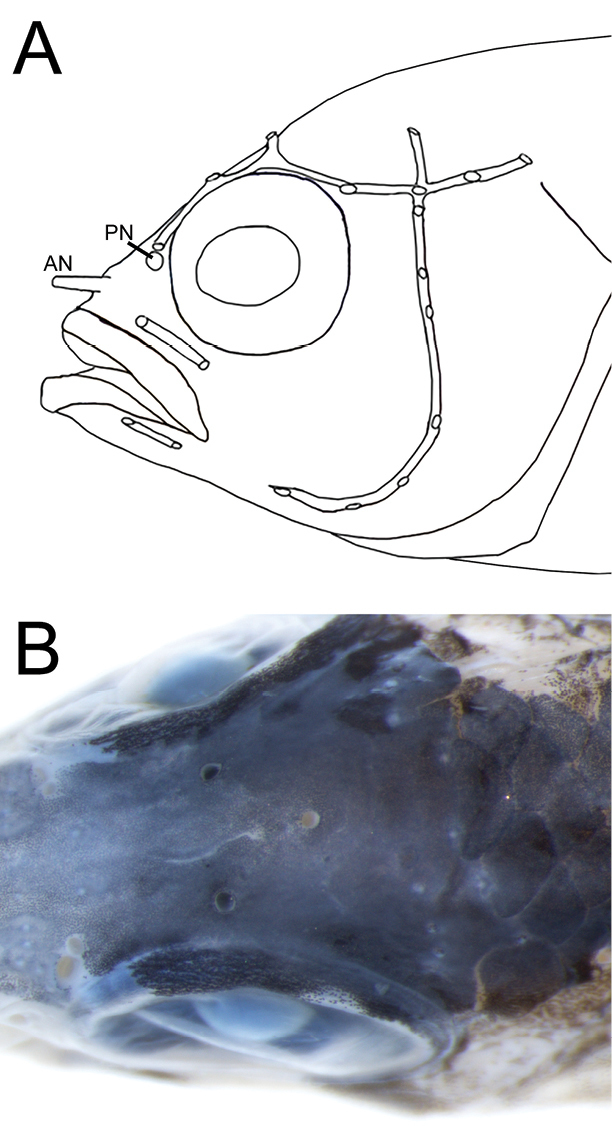
Cephalic sensory-canal pore system **A** composite pattern from entire type series **B** Supraorbital and median coronal pores, MUVS-V-137. Abbreviations: AN anterior naris, PN posterior naris.

**Figure 3.**
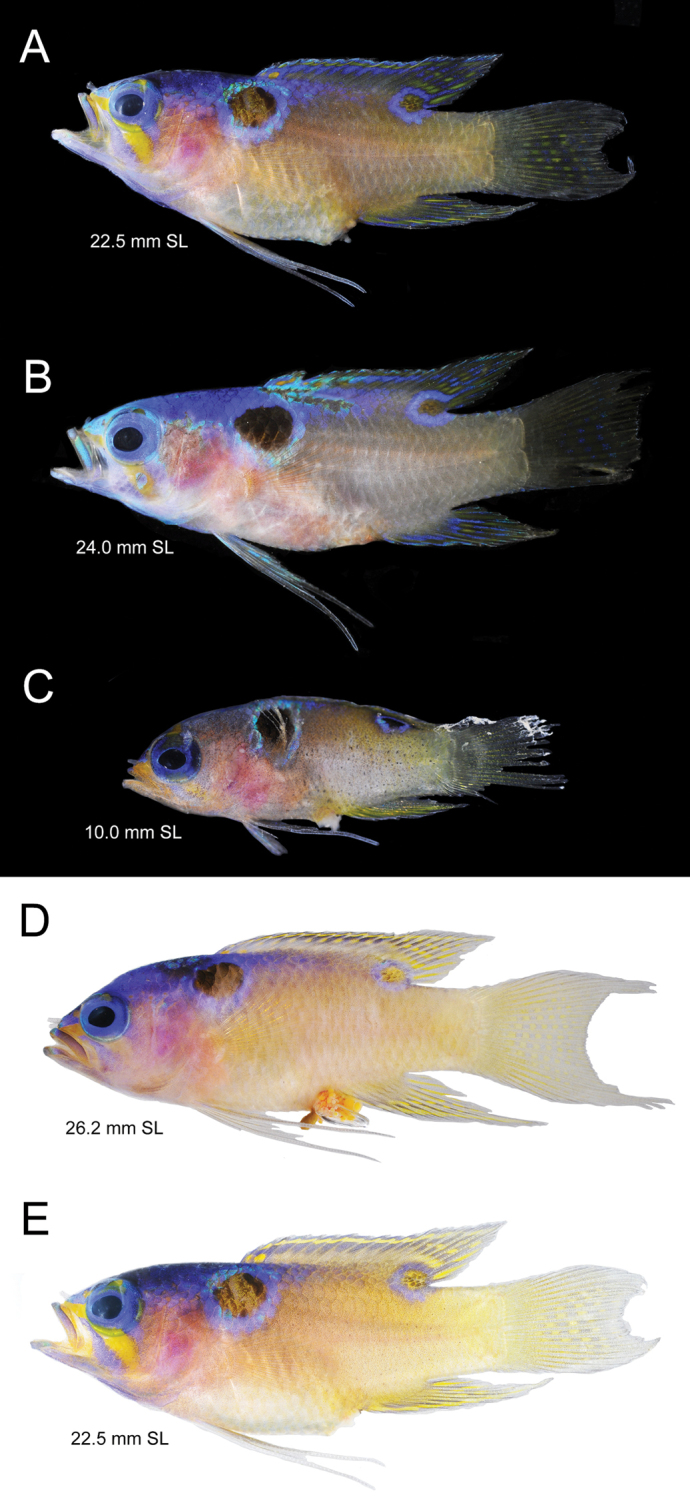
*Lipogrammaidabeli*, fresh specimens on dark (**A–C**) and light (**D, E**) backgrounds. **A**UF 240986 **B**MUVS-V-137 **C**UW 158096 **D**USNM 444940, holotype **E**MUVS-V-137.

**Figure 4.**
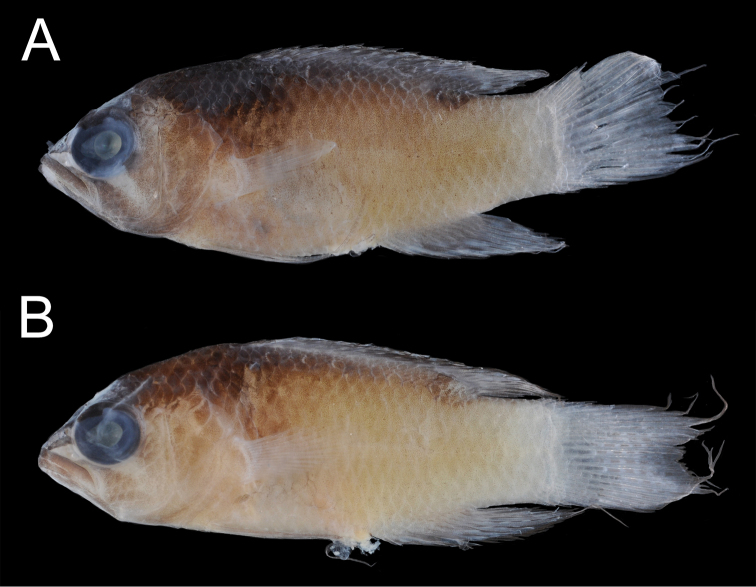
*Lipogrammaidabeli*, preserved **A**USNM 444940, holotype **B**UW 158090.

Figures 2 , 3 , 4 English: Blue-backed Basslet; Spanish: Cabrilleta de Dorso Azul

#### Type locality.

Roatan, Honduras, western Caribbean.

#### Holotype.

USNM 444940, 26.2 mm SL, tissue ROA17002, 165 m depth, station IDABEL17-01, reef slope off Halfmoon Bay, West End, Roatan, Honduras, 16.305557, -86.597669, *Idabel* Submersible, Luke Tornabene, D. Ross Robertson, Karl Stanley, 24 July 2017. **Paratypes.** Locality data same as that of holotype: UW 158090, 26.3 mm SL, tissue ROA17020, 137 m depth, station IDABEL17-05, Luke Tornabene, Rachel Manning, Karl Stanley, 29 July 2017; UW 158096, 10 mm SL, tissue ROA17026, 152 m depth, station IDABEL17-05, Luke Tornabene, Rachel Manning, Karl Stanley, 29 July 2017; MUVS-V-137, 24.0 mm SL, tissue ROA18041, 125–152 m depth, station IDABEL18-03, Luke Tornabene, Rachel Manning, Karl Stanley, 6 June 2018; UF 240986, 22.5 mm SL, tissue ROA18042, 125–152 m depth, station IDABEL18-03, Luke Tornabene, Rachel Manning, Karl Stanley, 6 June 2018.

#### Diagnosis.

A species of *Lipogramma* with pectoral-fin rays 15–16 (modally 16); gill rakers 18–20 total (10–11 elongate rakers plus 2–4 short, stout rudiments on lower limb, 3–4 elongate rakers plus 1–3 rudiments on upper limb); in life, body mostly yellow to tan with bright iridescent blue coloration on eye, dorsal portion of head, nape and dorsal portion of trunk beneath spinous-dorsal fin, oblique yellow bar from tip of snout to orbit and below eye, large, round, black blotch outlined with blue below anterior origin of dorsal fin, and dark ocellus outlined in blue with yellow or dark center at rear insertion of dorsal fin that extends onto body.

#### Description.

Counts and measurements of type specimens given in Table 1. Dorsal-fin rays XII, 9, last ray composite; anal-fin rays III, 7–8 (four specimens including holotype with 8, one with 7), last ray composite; pectoral-fin rays 15–16 (four specimens including holotype with 16, one with 15); pelvic-fin rays I,5; total caudal-fin rays 24 (13 upper, 12 lower), principal rays 17 (9 + 8), procurrent rays 6 (III+III), and an additional 2 unbranched rays (i+i) between principal and procurrent rays that are sometimes segmented; vertebrae 25 (10+15); pattern of supraneural bones, anterior dorsal-fin pterygiophores, and dorsal-fin spines 0/0/0+2/1+1/1/; ribs on vertebrae 3–10, epineurals visible in x-rays on vertebrae 1–13; gill rakers (counted from two specimens, UF 240986, MUVS-V-137) 18–20, upper limb with 3–4 elongate rakers plus 1–2 short rudiments, lower limb with 10–11 moderate-to-elongate rakers plus 2–4 small rudiments present only as nubs, all elongate rakers possess tooth-like secondary rakers, as in *L.evides* (Baldwin et al. 2016: fig. 3); pseudobranchia 6–7, filaments stout and highly branched; branchiostegals 6.

**Table 1.** Counts and measurements from type series. All measurements except SL are in % SL. Abbreviations: CP = caudal peduncle; PFO = pelvic-fin origin; P1 = pectoral fin; P2 = pelvic fin; DXII = twelfth dorsal-fin spine. Other caudal rays include i, a slender, flexible, non-spinous, sometimes segmented ray, and I, a spinous procurrent ray.

	USNM 444940	MUVS-V-137	UF 240986	UW 158090	UW 158096
	Holotype	Paratype	Paratype	Paratype	Paratype
Tissue number	ROA17003	ROA18041	ROA18042	ROA17020	ROA17026
SL (mm)	26.2	24	22.5	26.3	10
Dorsal-fin rays	XII, 9	XII, 9	XII, 9	XII, 9	XII, 9
Anal-fin rays	III, 8	III, 7	III, 8	III, 8	III, 8
Principal caudal rays	9+8	9+8	9+8	9+8	9+8
Other caudal rays	IIIi+iIII	IIIi+iIII	IIIi+iIII	IIIi+iIII	IIIi+iIII
Pectoral-fin rays	16	15	16	16	16
Gill rakers - upper limb	not counted	11+4 rudiments	10+2 rudiments	not counted	not counted
Gill rakers - lower limb	not counted	4+1 rudiment	3+3 rudiments	not counted	not counted
Head length	37.4	37.9	38.2	37.3	40.0
Eye diameter	13.0	14.6	14.2	12.9	2.0
Snout length	8.8	9.2	8.9	6.8	7.0
Depth at CP	20.2	17.9	19.1	19.0	17.1
Depth at PFO	36.3	30.8	35.6	33.4	35.5
Length P1 Fin	24.8	24.2	23.1	24.7	22.1
Length P2 Fin	50.4	45.4	44.9	46.4	35.0
Length DXII	14.8	13.7	12.0	14.1	13.0

Spinous and soft sections of dorsal fin confluent, several soft rays in posterior portion of fin forming slightly elevated lobe that extends posteriorly beyond base of caudal fin. Pelvic fin, when depressed, extending at least to origin of first soft ray of anal fin, and to origin of penultimate anal-fin ray in holotype. Dorsal profile from snout to origin of dorsal fin convex. Diameter of eye contained 2.8–3.0 times in head length. Pupil tear-shaped, with small aphakic space anteriorly. Scales extending anteriorly onto top of head, ending at a vertical just behind posterior margin of eye. Scales present on cheeks, operculum, and isthmus, absent on snout, jaws, and branchiostegals. Scales large and deciduous, missing on anterodorsal flank of several specimens. Approximately 22–25 lateral scales between shoulder and base of caudal fin (24 in holotype), 4–6 cheek rows, 11 rows across body from above anal-fin origin. Scales on nape and along dorsal midline with reduced or absent cteni, those on cheek and opercula lacking cteni. Fins naked except base of posteriormost dorsal-fin rays, which possess 1 or 2 small embedded scales. No modified lateral-line scales present on body, but faint indication of a lateral line present superficially in fresh photographs.

Margins of bones of opercular series smooth, opercle without spines. Single row of teeth on premaxilla posteriorly, broadening to 2 or 3 rows anteriorly, teeth in innermost row smallest, some teeth in outer row enlarged into small canines. Dentary similar with 3 or 4 enlarged teeth in outer row near symphysis. Vomer with chevron-shaped patch of teeth that extends posterolaterally nearly length of premaxilla, palatine with long series of small teeth.

Cephalic head pores arranged as in Fig. 2, with conspicuous pores present in infraorbital canal (2), dentary canal (2), supraorbital canal (2 pores above each eye plus one median coronal pore), preopercular canal (7), posttemporal canal (3), plus a single pore in canal above post-temporal canal in line with preopercle. Anterior naris in elongate tube above upper lip, posterior naris a wide opening with slightly raised rim immediately anterior to supraorbital canal.

Coloration in fresh specimens (Fig. 3): **Head**: top of snout, top of head, and nape bright blue; lower part of head yellow brown, with faint blue overtone; eye with bright blue iris, black pupil; top and bottom lips with pale blue overtone; yellow oblique bar, bordered with blue, extending from tip of snout to mid orbit and continuing below orbit towards lower corner of preopercle; opercle with lavender hue from bright red gills and blood vessels visible through gill cover. **Trunk**: yellowish to yellow-brown, paler and sometimes with faint bluish cast on isthmus and abdomen; large, eye-size round black blotch on upper back under the origin of dorsal fin, surrounded by thin bright blue ring; blue coloration on nape extends along the upper back to end of spinous dorsal. **Spinous dorsal fin**: bright blue anteriorly, fading to blue-grey posteriorly on basal half; outer edge blue-grey, with thin submarginal yellow stripe formed by series of close-set, horizontally elongate yellow spots; row of yellow spots (each on and just behind a spine), beginning at base of the first 2–3 spines, then continuing as row along other 1/3 of the fin**. Soft dorsal fin**: rays blue-grey anteriorly, with the last several posterior rays blue; upper margin whitish-blue, with a submarginal row of vertically elongate, inter-radial yellow spots, and 3–4 rows of vertically oval, inter-radial yellow spots, those at rear forming thin, yellow lines along the membranes between last 2 or 3 rays; pupil-sized, round, yellow-brown blotch containing darker scales ringed with bright blue at rear insertion of fin, with half of blotch covering base of last 6 or 7 rays and half on upper back. **Anal fin**: bluish grey, with brownish cast on scaled base of fin; 3 or 4 irregular rows of yellow spots along fin elements, central spots oval, basal and outer spots forming streaks along fin elements; outer margin of fin whitish-blue. **Caudal fin**: base translucent yellow; center of fin with rows of yellow spots along fin rays; outer part of fin translucent, with thin whitish-blue rear margin; vertical rows of pale blue spots on rays in center of fin. **Pectoral fins**: pectoral rays tinged with yellow, membranes translucent; base of fin paler than adjacent body. **Pelvic fins**: pale bluish grey, with elongate yellowish spots along fin rays, that yellow coloration strongest at base of fin.

Juvenile coloration: Coloration of the single small juvenile is essentially the same as in the adult, except that the posterior portion of the body is more noticeably yellow, the entire center of the anal fin, and, apparently, much of the soft dorsal and caudal fins are yellow, and the dark center of the ocellus at the lower rear corner of the dorsal fin is solid black.

Comments about live coloration: As can be seen in the video of the holotype being captured (https://doi.org/10.5281/zenodo.1334518, or https://zenodo.org/record/1334518#.W89WsSPMyFU), live fish have the upper third of the head and body bright blue, the lower 2/3 yellow, and a prominent large round black blotch on the shoulder.

Color in preservation (Fig. 4): Overall pigmentation pattern largely similar to that of fresh individuals, except iridescent blue coloration replaced by dark brown pigment; oblique bar on head below eye pale in preservation versus yellow in life; dark ocelli below spinous and soft dorsal fins less distinct, with no clearly defined ring or margin; background body coloration darkest on head and below origin of spinous dorsal fin, gradually fading from brownish-tan anteriorly to pale-yellow posteriorly.

#### Distribution.

**Figure 5.**
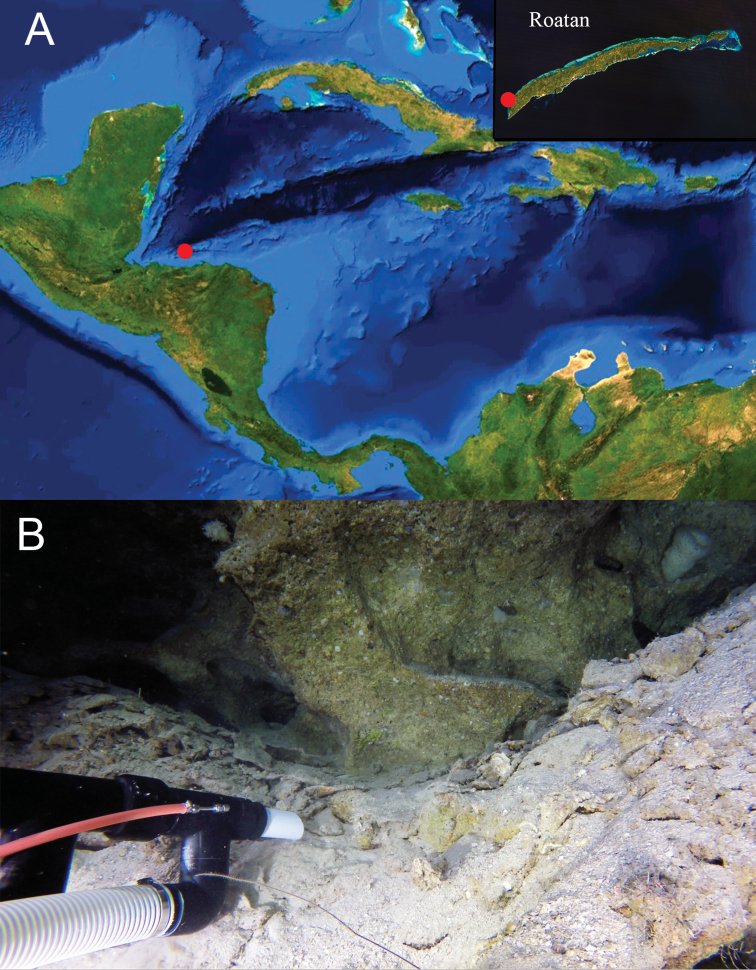
Type locality. **A** Halfmoon Bay, Roatan, Honduras. **B** Habitat where holotype was collected 165 m depth. The rock pile immediately in front of the suction tube is where the fish was sheltered. Maps courtesy of NASA.

Known only from specimens collected off Roatan, Honduras (Fig. 5).

#### Habitat.

The species was frequently observed in the mid-to-upper rariphotic zone between 122–165 m depth, in or around small rock crevices, rock piles, or caves situated on steep limestone walls covered with coarse sediment and fine rubble composed of dead sections of the green macroalga *Halimeda* (Fig. 5). A video showing the collection of the holotype is available online (https://doi.org/10.5281/zenodo.1334518, or https://zenodo.org/record/1334518#.W89WsSPMyFU).

#### Etymology.

The specific epithet *idabeli* refers to the *Idabel* submersible, which was used to collect the type series, and recognizes of the efforts of its owner-designer and pilot Karl Stanley and engineer Thomas Trudel, who made these and other collections of fishes possible by constructing a fish-catching system that converted *Idabel* from an observation-only vessel to one capable of collecting scientific specimens. The name *idabeli* is to be treated as a patronym (adjective) formed from the female name Idabel. The generic name *Lipogramma* is feminine and is formed from *lipo* (without) and *gramma* (a line of text, feminine), referring to the absence of a well-developed lateral line. The common name Cabrilleta de Dorso Azul (Blue-backed Basslet in English) refers to its distinctive coloration.

#### Comparisons.

**Figure 6.**
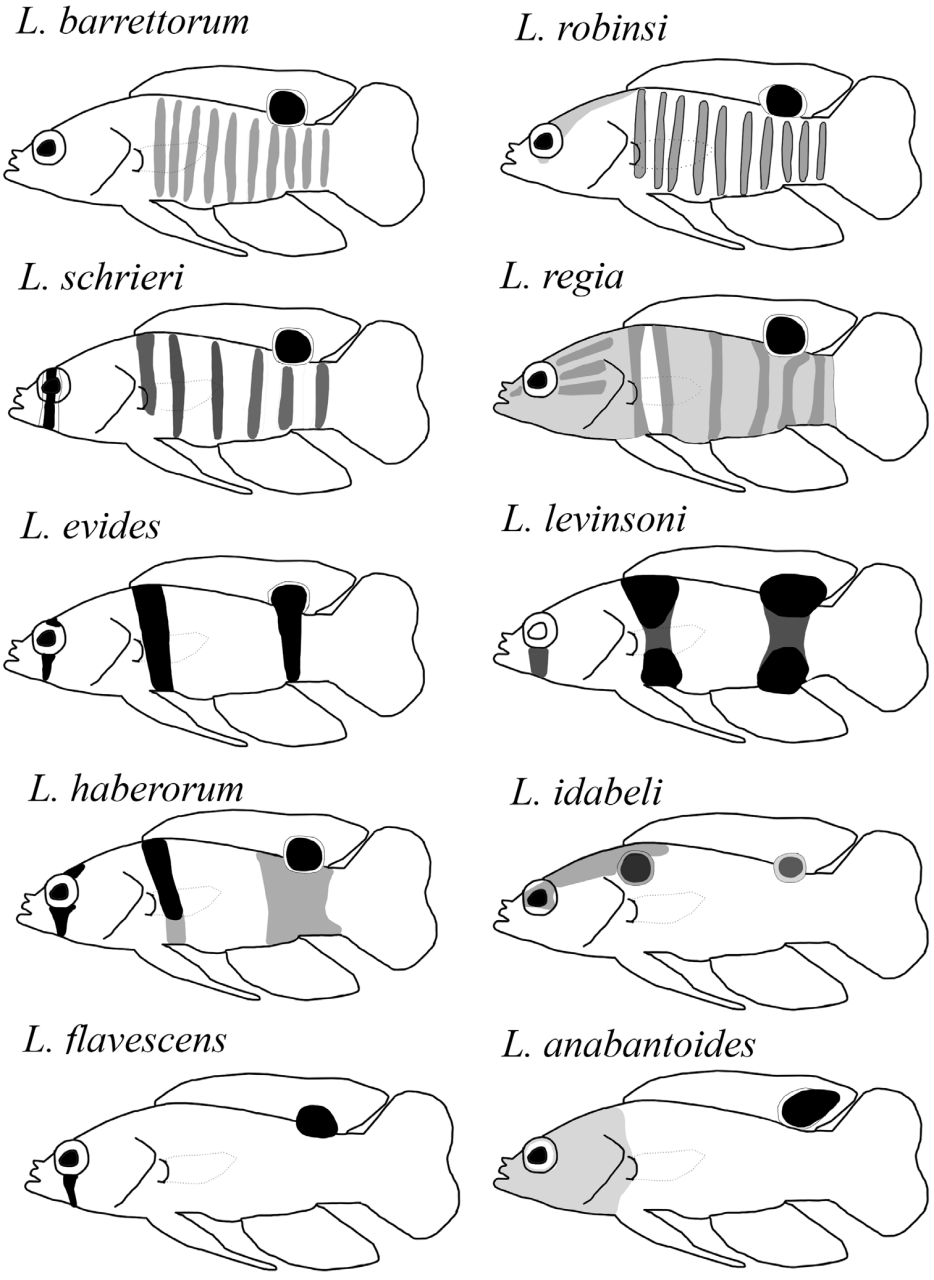
Schematic showing the barring and shading patterns of the ten species of *Lipogramma* that possess a dark ocellus on the posterior portion of the dorsal fin.

*Lipogrammaidabeli* is easily distinguished from all other species of *Lipogramma* by the bright blue coloration on the head and eye, and by the pair of blue-margined ocelli below the anterior origin of the dorsal fin and at the posterior insertion of the dorsal fin. There are only two other species of *Lipogramma* in which the head is a markedly different color than the body; *L.klayi* and *L.anabantoides* both have rose, pink, or purple heads with tan or yellow bodies. No other known species has bright blue coloration on the dorsal portion of the head, nape, and dorsal portion of the trunk. All species of *Lipogramma* except *L.klayi*, *L.rosea*, and *L.trilineata* possess an ocellus on the posterior portion of the dorsal fin, but the pattern of barring and shading on the head and body differ among all of those species (Fig. 6). In addition to the differences, none have the ocellus with a blue margin except *L.idabeli*. Furthermore, in *L.idabeli*, the ocellus varies from having a dark center as a juvenile to a yellow or dusky center as an adult, whereas in the other species, the ocellus is always black. The absence of prominent vertical barring on the body distinguishes *L.idabeli* from *L.robinsi*, *L.barretorum*, *L.haberorum*, *L.evides*, *L.levinsoni*, *L.schrieri*, and *L.regia* (Fig. 6). *Lipogrammaidabeli* has more total gill rakers (18–20) than all other species except *L.evides* (19–22), *L.klayi* (19), and *L.levinsoni* (17–20); all other species have 16 or fewer. The combination of XII, 9 dorsal-fin elements and III, 8 anal-fin elements present in *L.idabeli* is shared among most species in the genus, except *L.rosea* (XI, 6 and III, 6), *L.trilineata* (XII, 10 and III, 7), and *L.anabantoides* (XIII, 8 and III, 8).

#### Phylogenetics and eco-evolutionary relationships.

**Figure 7.**
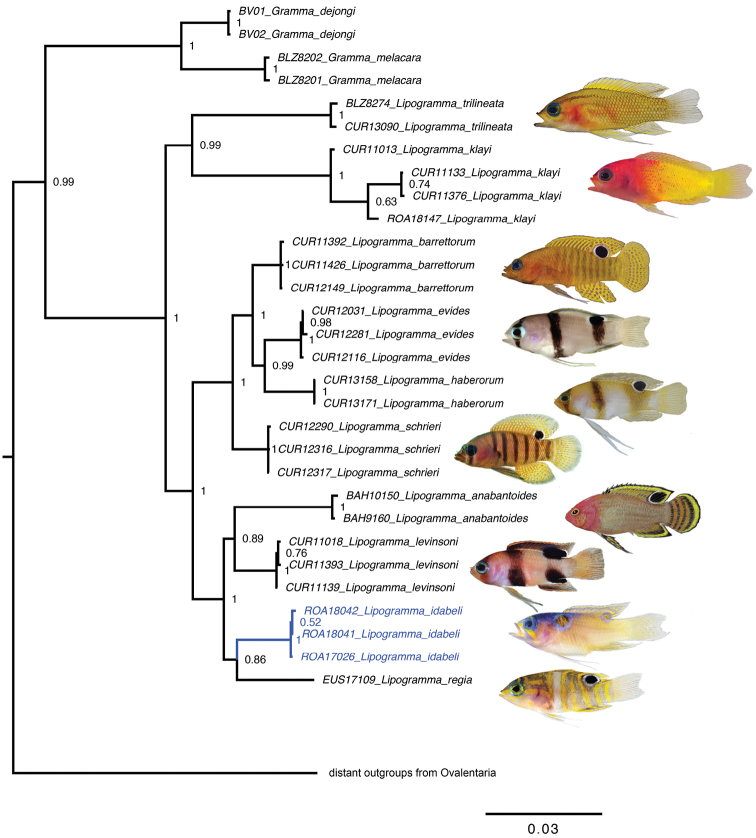
Bayesian inference molecular phylogeny of *Lipogramma*. Numbers at nodes are posterior probabilities. Photographs and illustrations by CC Baldwin, DR Robertson, L Tornabene, RG Gilmore, and CR Robins.

Coloration unambiguously diagnoses *L.idabeli* and supports its recognition as a distinct species. Molecular data from the ten species of *Lipogramma* for which tissue samples were available also support this distinction. The molecular phylogenies from the Bayesian and Maximum Likelihood analyses (Fig. 7; Suppl. material 1: Figure S1), which were identical in topology to that from the BP&P coalescent species-tree analysis, show that the three specimens of *L.idabeli* form a monophyletic group with strong support (1.0 posterior probability; 100 bootstrap). The BP&P species delimitation analysis had overwhelming support for a 10-species model (posterior probability 0.997) versus models with fewer or more species, indicating perfect congruence between morphological and molecular delimitation approaches.

Our analyses show *L.idabeli* as part of a well-supported clade containing *L.regia*, *L.levinsoni*, and *L.anabantoides*, with the relationships within this clade being less resolved. All of these species occur from the mid-to-upper rariphotic zone and shallower (20–165 m). Similar to our observations of species of *Lipogramma* occurring off Curacao and other localities in the Caribbean (Baldwin et al. 2016, 2018), species off Roatan appear to be partitioning the reef by depth and microhabitat association. The known depth range for *L.idabeli* off Roatan, based on collected specimens and visual observations, is 122–165 m. At Roatan, its depth range broadly overlaps with that of *L.levinsoni*, but the two species occupy very different microhabitats. *Lipogrammalevinsoni* is typically found hovering around and above limestone rubble and small cobble habitats on gradual slopes, whereas *L.idabeli* is found around larger rocks, caves, and outcroppings on steeper slopes and vertical walls. In addition to *L.idabeli* and *L.levinsoni*, other nominal species of *Lipogramma* observed at Roatan include *L.klayi*, *L.evides*, and *L.flavescens*. Like *L.idabeli*, *L.klayi* also occurs around steep walls but at depths considerably shallower than *L.idabeli* (~65–120 m versus 122–165 m), where the reef wall is generally covered with more extensive growth of *Halimeda*, encrusting sponges, gorgonians, and other sessile habitat-forming organisms. Both *L.flavescens* and *L.evides* were observed deeper than *L.idabeli* at 213–250 m, with *L.evides* usually occurring on gradual rocky slopes with a heavy layer of cobbles (similar to the habitat of *L.levinsoni*), and *L.flavescens* found out in the open on bottoms of coarse sand with small, low, scattered piles of rock and rubble, far from the wall.

## Supplementary Material

XML Treatment for Lipogramma idabeli

## Appendix 1

**Table A1.** DNA voucher specimens, GenBank numbers, and GenSeq nomenclature for new sequences generated in this study.

**Catalog number**	**Tissue number**	**Type**	**Species**	**GenBank COI**	**GenBank TMO-4c4**	**GenBank Rag1**	**GenBank Rhodopsin**	**GenSeq Designation**
MUVS-V-137	ROA18041	paratype	* Lipogramma idabeli *	MK227831	MK227821	MK227827	MK227824	genseq-2
UF 240986	ROA18042	paratype		MK227830	MK227822	MK227826	MK227825	genseq-2
UW 158096	ROA17026	paratype		MK227829	MK227820	MK227828	MK227823	genseq-2

## Supplementary materials

Supplementary material 1 Figure S1 Data type: statistical data Explanation note: Maximum likelihood inference molecular phylogeny of *Lipogramma*. Numbers at nodes are bootstrap support values. This is an open access article distributed under the terms of the Creative Commons Attribution License (CC BY 4.0), which permits unrestricted use, distribution, and reproduction in any medium, provided the original author and source are credited.Luke Tornabene, D. Ross Robertson, Carole C. Baldwin
