# Supplementary figures and images for: A new species of Lipogramma from deep reefs of Roatan, Honduras (Teleostei, Grammatidae)
Source: Zookeys. 2018 Dec 19;(809):79–95. doi: 10.3897/zookeys.809.29280 (PMC6321867; doi:10.3897/zookeys.809.29280)

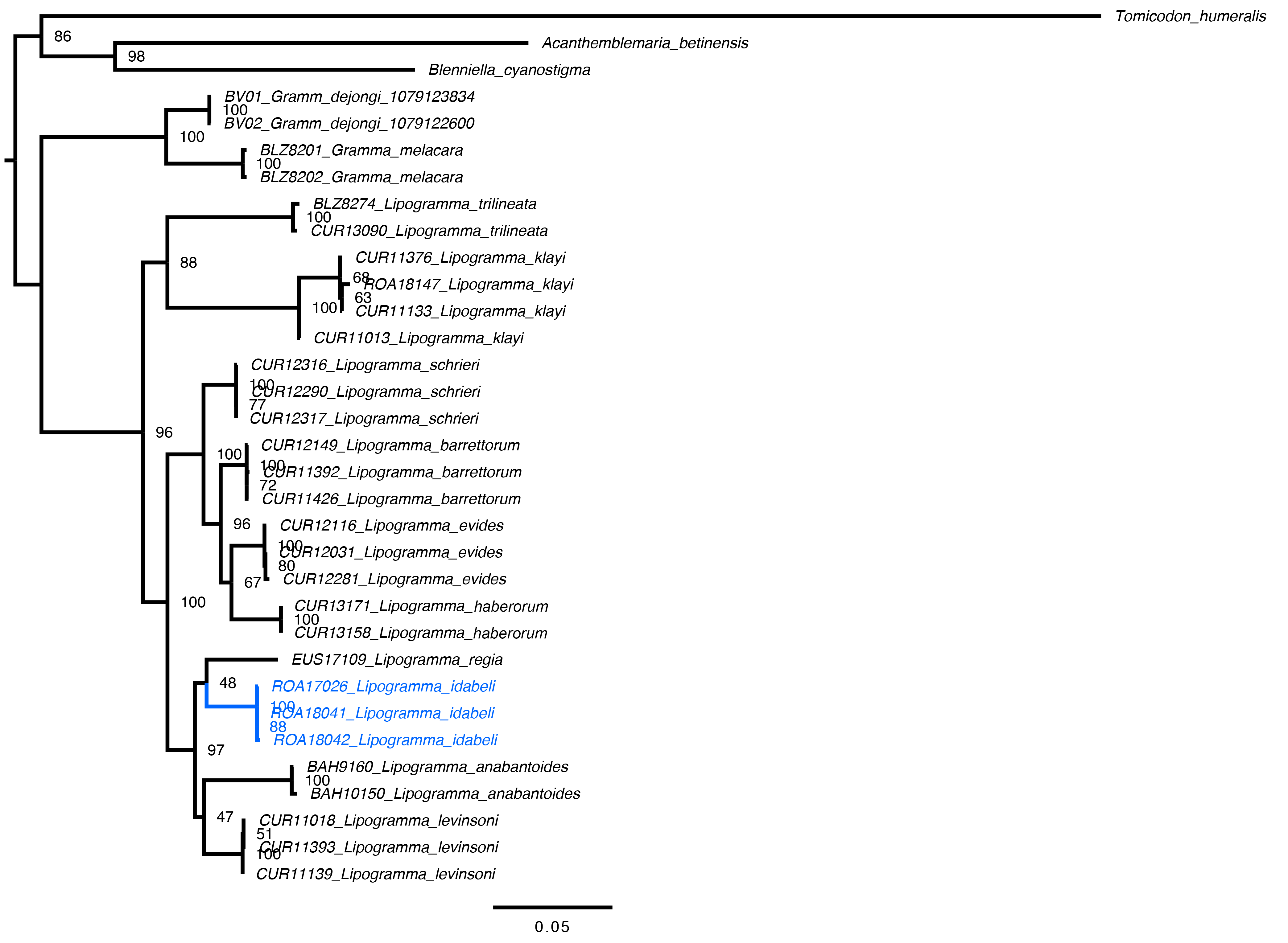

Supplement: Supplementary material 1 — Figure S1 [file zookeys-809-079-s001.tif]
